# Stereotactic Radiosurgery for Brain Metastases in Patients With a Heterozygous Germline Ataxia Telangiectasia Mutated Gene

**DOI:** 10.7759/cureus.37712

**Published:** 2023-04-17

**Authors:** Jennifer C Hall, Steven D. Chang, Melanie H Gephart, Erqi Pollom, Santino Butler

**Affiliations:** 1 Radiation Oncology, Stanford University School of Medicine, Stanford, USA; 2 Neurosurgery, Stanford University School of Medicine, Stanford, USA

**Keywords:** treatment outcomes of cyberknife, stereotactic radiosurgery srs, brain radiation necrosis, atm mutation, brain stereotactic radiosurgery

## Abstract

Germline mutations in the ataxia telangiectasia mutated (*ATM*) gene are associated with increased radiation sensitivity. Present literature lacks consensus on whether patients with heterozygous germline *ATM* mutations may be at greater risk of radiation-associated toxicities when treated with radiation therapy (RT), and there is little data considering more modern and conformal RT techniques such as stereotactic radiosurgery (SRS). Our report presents two cases of patients with heterozygous germline *ATM* mutations treated with SRS for brain metastases. One patient developed grade 3 radiation necrosis (RN) of an irradiated 16.3 cm^3^ resection cavity, but did not develop RN at other sites of punctate brain metastases treated with SRS. Similarly, the second report describes a patient who did not develop RN at any of the 31 irradiated sites of sub-centimeter (all ≤5 mm) brain metastases. The described cases demonstrate that some patients with germline *ATM* variants can safely undergo SRS for smaller brain metastases; however, clinical caution should be considered for patients with larger targets or a history of prior radiation toxicity. Given these findings and the lingering uncertainty surrounding the degree of radiosensitivity across *ATM* variants, future research is needed to determine whether more conservative dose-volume limits would potentially mitigate the risk of RN when treating larger brain metastases in this radiosensitive population.

## Introduction

Homozygous germline mutations in the ataxia telangiectasia mutated (ATM) gene are associated with cerebellar degeneration, telangiectasia, immunodeficiency, and cancer susceptibility, the constellation of ataxia telangiectasia (AT) syndrome [1-2]. *ATM* is a tumor suppressor gene, which encodes for a protein involved in the coordination and regulation of DNA double-strand breaks, such as those occurring in response to ionizing radiation [3]. Accordingly, patients with homozygous germline *ATM* mutations can develop severe toxicity with radiation for cancer treatment [4-5].

The presence of heterozygous germline *ATM* mutations may be associated with a more moderate increase in radiation sensitivity, though the clinical relevance of this radiosensitivity in vivo has not been thoroughly explored and there are conflicting reports regarding the relative risk of radiation-associated toxicities in this patient population [6-9]. There is limited understanding of radiation-associated toxicities across various cancer disease sites, normal uninvolved organ tissues, and radiation treatment modalities in patients with heterozygous germline *ATM* variants. Specifically, there is a paucity of data surrounding the safety of stereotactic radiosurgery (SRS) for these patients with brain metastases.

SRS delivers highly conformal, ablative radiation doses and is commonly used to treat brain metastases. A notable late effect of SRS for brain metastases is radiation necrosis (RN), which has an incidence of 5-25%, and is associated with the size of metastases [10]. It is unclear whether the presence of heterozygous germline *ATM* variants, compared to *ATM* wildtype, may confer greater risk of RN after SRS.

Here, we present two cases of patients with heterozygous germline *ATM* mutations treated with SRS for brain metastases. To our knowledge, there are no previously described cases of SRS for brain metastases in patients with germline *ATM* mutations.

## Case presentation

Case one

**Figure 1:**
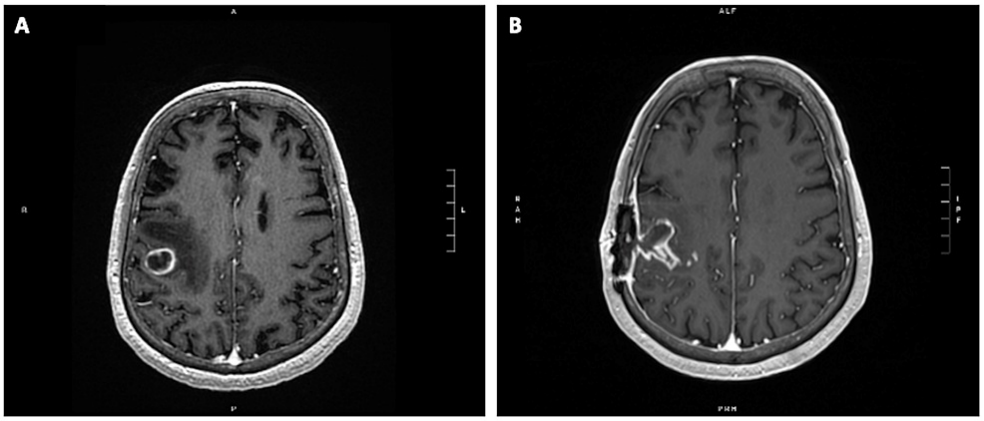
Preoperative and postoperative imaging of the posterior right frontal lobe brain metastasis (A) Preoperative T1 MRI with contrast showed the 1.8 cm intact brain metastasis with surrounding edema extending from precentral gyrus to the frontal subcortical white matter with mild mass effect. (B) A postoperative T1 MRI demonstrated postsurgical changes. This postoperative MRI was fused with a CT scan for treatment planning.

A 61-year-old male (Patient A) with a heterozygous germline *ATM* mutation (c.7417del (p.Cys2473Alafs*3)) and multiple prior malignancies, presented with newly diagnosed lung adenocarcinoma and left-sided weakness, with brain MRI demonstrating a solitary 1.8 cm right frontal lobe metastasis (Figure 1). He underwent gross total resection (GTR) of this right frontal lobe brain metastasis with pathology confirming malignancy of lung origin.

**Figure 2:**
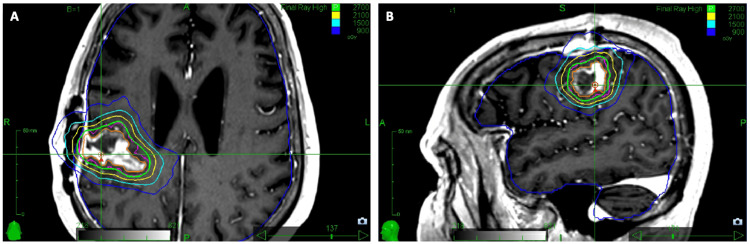
Cavity SRS treatment planning (A) Axial cut through the GTV contoured in orange and PTV contoured in pink. The PTV was created by adding a 2 mm isometric expansion to the GTV. (B) Sagittal view. SRS: stereotactic radiosurgery; GTV: gross tumor volume; PTV: planning target volume

The patient was treated with cavity SRS one month after GTR to improve local control [11]. A planning target volume (PTV) of 16.26 cm^3^ was contoured as the resection cavity plus a 2 mm isometric expansion [12]. The PTV was treated to a prescribed dose of 27 Gy in three fractions to the 73% isodose line (IDL) (Figure 2). The single fraction equivalent dose with an assumed alpha/beta ratio of 10 (SFED_10_) was 18.19 Gy. The SRS plan achieved a conformity index (CI) of 1.14 and PTV coverage >95%.

**Figure 3:**
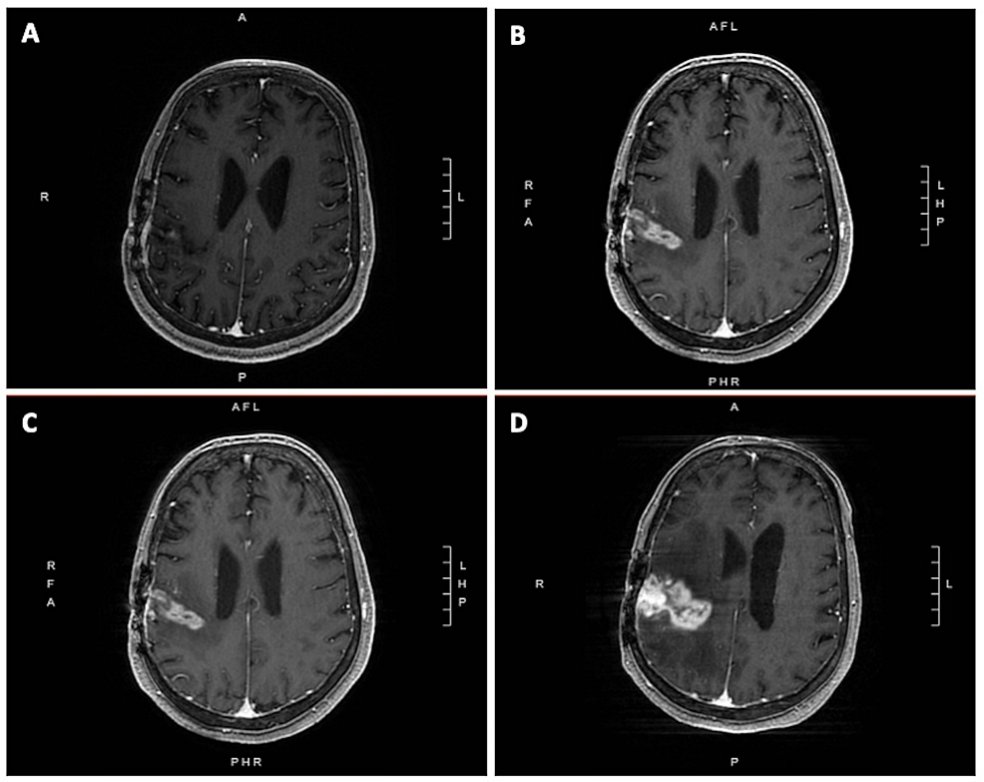
Surveillance imaging Serial T1 MRI with contrast reveals the gradual worsening of radiation necrosis at the treated resection cavity from approximately (A) three months, (B) six months, (C) 11 months, and (D) 13 months after completion of cavity SRS. SRS: stereotactic radiosurgery

Patient A tolerated SRS without acute complication and he continued with regular imaging surveillance every three months. Six months after SRS, MRI of the brain revealed two new, punctate intracranial metastases in the left parietal lobe and left superior cerebellar hemisphere; the previously treated resection cavity appeared with new ill-defined enhancement along the surgical margins and medial aspect of the resection cavity which was thought to be treatment-related changes (Figure 3A).

**Figure 4:**
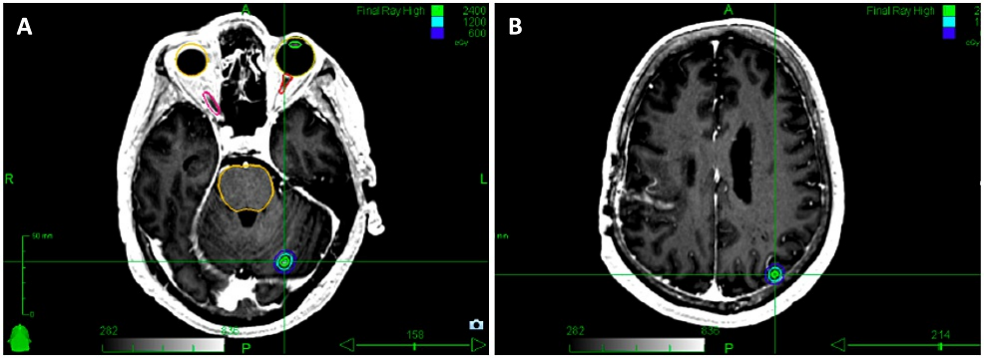
SRS treatment plan for two intact brain metastases, left cerebellar and left parietal lobe (A) Axial view of the left cerebellar brain metastasis treated to 24 Gy in one fraction with critical structures also delineated to minimize dose to these areas. (B) Axial view of the left parietal brain metastasis also treated to 24 Gy in one fraction. SRS: stereotactic radiosurgery

The two new enhancing metastases were treated with an additional course of SRS to a prescribed dose of 24 Gy in one fraction each to the 74% IDL. These intact metastases were treated with a 0 mm margin for PTV. The two metastases were treated in a single SRS plan with a CI of 1.26 and 100% PTV coverage (Figure 4). The left parietal lesion PTV was 0.02 cm^3^; the left superior cerebellar lesion PTV was 0.04 cm^3^.

His second course of SRS was well tolerated without acute toxicity and the patient continued on a three-month surveillance schedule. Following SRS, he was subsequently started on adjuvant carboplatin (target AUC 5)/pemetrexed (500 mg/m^2)^, but this was discontinued after one cycle due to grade 4 pancytopenia, and further chemotherapy was held. 

Six months following his second course of SRS (13.5 months since completion of his initial post-operative cavity SRS), Patient A was admitted for altered mental status and urinary retention. Repeat MRI of the brain revealed evidence of worsening RN at the right frontal resection cavity, as well as two new punctate lesions at the paramedian superior frontal gyrus and right inferior cerebellum (Figure 3D). 

Given the radiographic confirmation of symptomatic RN, Patient A was started on a steroid taper and bevacizumab 5 mg per kg every two weeks for three planned cycles. Between the first and second cycles of bevacizumab, Patient A underwent a third course of SRS for the two new metastases noted on his recent MRI.

**Figure 5:**
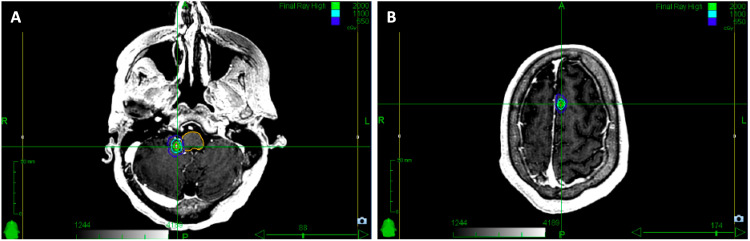
SRS treatment plan for two additional intact brain metastasis, right cerebellar and left frontal lobe (A) Axial view of the right cerebellar brain metastasis treated to 20 Gy in one fraction with critical structure also delineated to minimize dose to these areas. (B) Axial view of the left frontal brain metastasis also treated to 20 Gy in one fraction. SRS: stereotactic radiosurgery

The two lesions were treated with a single SRS plan to a prescribed dose of 20 Gy in one fraction to the 67% IDL. The plan achieved a CI of 1.51 and 100% PTV coverage (Figure 5). The PTVs were 0.04 cm^3^ and 0.09 cm^3^ for the frontal gyrus and right cerebellar lesions, respectively.

At the time of his most recent follow-up, Patient A was three-months status post completion of his third course of radiotherapy and three cycles of bevacizumab (17 months since completion of his initial post-operative cavity SRS), and all neurologic and cognitive symptoms were resolved. His most recent MRI of the brain was grossly stable with similar appearing enhancement at the later aspect of the resection cavity thought to be evolving post-treatment effect status post three cycles of bevacizumab.

Case two

A 64-year-old male (Patient B) with a heterozygous germline-*ATM* mutation (c.2413C>T (p.Arg805*)) and remote history of right-sided breast cancer and synchronous bilateral non-small cell lung cancers (NSCLC) presented after numerous, bilateral supra- and infratentorial brain lesions were noted on surveillance imaging for a known, stable right vestibular schwannoma. The ring-enhancing lesions, located largely at the gray-white junction, were felt to be consistent with brain metastases, and the patient was recommended to undergo SRS to 31 total sites of intracranial disease.

**Figure 6:**
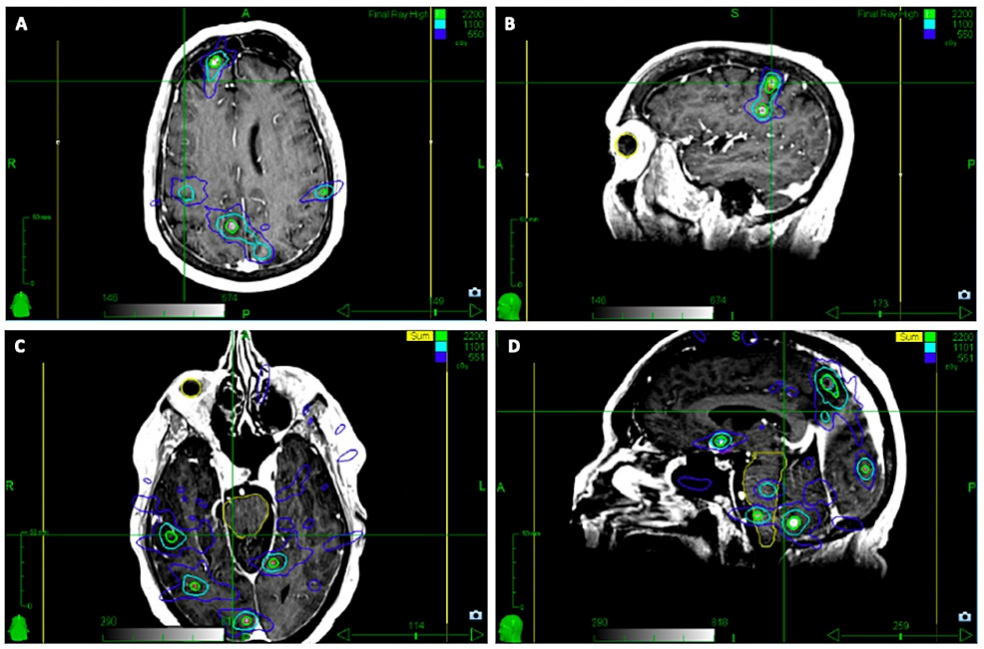
SRS treatment plan for the treatment of 31 intact, punctate brain metastases The brain metastases were treated to 22 Gy in one fraction each across two plans. (A, B) The first plan targeted 16 lesions with a total PTV of 2.89 cubic centimeters; (C, D) the second plan targeted 15 lesions with a total PTV of 2.23 cubic centimeters. The cumulative PTV across both plans was 5.12 cubic centimeters. The target lesions were contoured and critical structures including eyeballs and brainstem were delineated (yellow). SRS: stereotactic radiosurgery; PTV: planning target volume

The 31 lesions ranged from 2-5 mm in maximum diameter and were treated to a prescribed dose of 22 Gy in one fraction each to the 76% IDL. The treatments were delivered over two consecutive days and contoured in two discrete treatment plans: the first plan targeted eight bilateral frontal lobe metastases and seven bilateral parietal lobe metastases, as well as one left insular metastasis for a total of 16 lesions and a total treatment volume of 2.89 cm^3^. This plan achieved a CI of 1.42 and PTV coverage >95% (Figure 6A). The second plan targeted 15 total brain metastases for a total treatment volume of 2.23 cm^3^: four lesions in the right temporal lobe, one lesion in the right occipital lobe, eight lesions in the cerebellum, and two lesions located in the brain stem. This plan achieved a CI of 1.66 and PTV coverage >95% (Figure 6B).

Patient B tolerated SRS without acute adverse effects and proceeded with follow-up imaging at three-month intervals. He received six cycles of adjuvant carboplatin (target AUC 5)/ pemetrexed (500 mg/m^2)^ followed by maintenance pemetrexed (500 mg/m^2)^. He maintained stable intracranial disease and was without clinical or radiographic evidence of RN at the time of his last brain imaging, 16 months after completion of SRS.

Patient B ultimately developed continued thoracic and osseous disease progression which, despite multiple lines of systemic therapy, contributed to worsened cancer-related pain and shortness of breath. Eighteen months after completion of SRS for his 31 brain metastases, the patient died of respiratory failure secondary to his advanced progressive disease.

## Discussion

Although heterozygous germline *ATM* mutations may confer increased radiosensitivity, it is unclear whether this results in increased risk of radiation-associated toxicities [4]. Previous studies note that when treated with RT for breast cancer, patients with heterozygous *ATM* variants may have an increased risk of radiation-induced subcutaneous late tissue effects [13]; whereas, other reports find no evidence of excess radiation-associated toxicity with breast radiotherapy [14]. Still other data suggest that specific *ATM* single nucleotide polymorphisms (SNPs), such as rs1801516 (c.5557G>A, pAsp1853Asn), are associated with radiation toxicity in breast and prostate cancer patients; however, meta-analyses examining this question have also been conflicting [15-16]. The uncertainty surrounding the clinical implications of heterozygous germline* ATM* mutations is further complicated by the development of more conformal radiotherapy techniques, including SRS. Here, we described two patients with heterozygous germline *ATM* mutations who received SRS for brain metastases.

The potential toxicity relevant to SRS for brain metastases is radiation necrosis. Longstanding studies have shown increased incidents of RN with larger SRS targets, based on maximum diameter [10]. Indeed, Patient A developed grade 3 RN at his irradiated resection cavity, which had a maximum diameter on postoperative imaging of 38 mm. Patient A did not develop RN associated with his other treated lesions, which were much smaller. Similarly, Patient B did not develop RN with any of his 31 treated brain metastases, which were all no larger than 5 mm in maximum diameter.

Shaw et al. identified SRS targets measuring 31-40 mm in maximum diameter had a 16 times higher risk of developing CNS toxicities, including RN. However, for patient A, we would anticipate this increased risk to be partially mitigated given the use of multi-fraction SRS (MF-SRS) with 27 Gy in three fractions; as data from Minniti et al. showed that for lesions >3 cm, the rate of symptomatic grade 2-3 RN was 2.9% with MF-SRS versus 8.6% with single fraction-SRS [17-19]. Thus, there is a possibility that patient A had increased susceptibility to radiation toxicity. Interestingly, Patient A had a history of three aggressive cancers, as well as a history of prior grade 4 toxicity after prior stereotactic body radiation therapy (SBRT) to the pancreas (ie. hemorrhagic shock secondary to superior mesenteric artery pseudoaneurysm bleed, which occurred eight months after SBRT, 40 Gy in four fractions), which altogether would suggest the potential for both a high-risk *ATM* genotype and phenotype. This highlights that the degree of patient susceptibility to radiation late effects could potentially vary both by specific *ATM* mutations, as well as by different individual penetrance and expressivity of these mutations. In fact, it has been shown that the risk and aggressiveness of breast cancer oncogenesis do vary with *ATM* penetrance and expressivity [9,20]; yet to our knowledge, it remains unknown whether this concept extends to radiosensitization phenotype and late treatment effects. If so, this may help to explain some of the confounding evidence seen in prior literature on this subject.

This then raises the question of whether a lower or more conservative dose-volume metric should be considered in patients with germline *ATM* mutations, particularly those with a demonstrated history of toxicity following radiation therapy. Individual genetic risks can in theory lead to inaccuracies of traditional biological effective dose calculations and alpha/beta ratio assumptions as these do not take factors such as *ATM*-related radiation sensitivity into account. These cases also underscore the importance of the emerging investigation into personalized genetic risk scores for predicting radiation toxicity.

In summary, the described cases demonstrate that some patients with germline *ATM* variants can safely receive SRS for sub-centimeter brain metastases. However, it may be reasonable to exercise caution for those with relatively larger targets or a history of prior radiation toxicity. 

## Conclusions

In patients with heterozygous germline *ATM* mutations, SRS may be safe for smaller brain metastases; however, the relative size of brain targets and patient history of prior radiation toxicity should be factors that are strongly considered when weighing the decision for treatment. This report adds to the scant literature concerning radiation-associated toxicities in patients with heterozygous germline *ATM* mutations, and particularly explores late-treatment effects in this population following highly conformal treatment with SRS for brain metastases. Future research is needed to determine whether a more conservative dose-volume interplay in the treatment of brain metastases for patients with *ATM* germline mutations may help reduce the risk of RN after SRS in this radiosensitive population.
